# Enhanced transfection of a macromolecular lignin-based DNA complex with low cellular toxicity

**DOI:** 10.1042/BSR20181021

**Published:** 2018-11-30

**Authors:** Yoon Khei Ho, Dan Kai, Geraldine Xue En Tu, G. Roshan Deen, Heng Phon Too, Xian Jun Loh

**Affiliations:** 1 Department of Biochemistry, National University of Singapore, 8 Medical Dr, Singapore 117596; 2 Institute of Materials Research and Engineering, A*STAR (Agency for Science, Technology and Research), 3 Research Link, Singapore 117602; 3 Soft Materials Laboratory, Natural Sciences and Science Education, National Institute of Education, Nanyang Technological University, Singapore; 4 Department of Materials Science and Engineering, National University of Singapore, 9 Engineering Drive 1, Singapore 117576

**Keywords:** cationic polymer, Gene delivery, lignin copolymer, transfection enhancer

## Abstract

Cationic polymers remain attractive tools for non-viral gene transfer. The effectiveness of these vectors rely on the ability to deliver plasmid DNA (pDNA) into the nucleus of cells. While we have previously demonstrated the potential of Lignin-PGEA-PEGMA as a non-viral gene delivery vector, alterations of cellular phenotype and cytotoxicity were observed post transfection. The present study aims to explore transfection conditions for high efficiency and low toxicity of the Lignin-PGEA-PEGMA based gene delivery system. Cellular toxicity was significantly reduced by using the centrifugation protocol, which enables rapid deposition of DNA complexes. Replacement of media post centrifugation resulted in minimal exposure of cells to excess polymers, which were toxic to cells. At an optimized DNA amount (500–750 ng) and molar ratios of nitrogen (N) in polymer to phosphate (P) in pDNA (N/P = 30–40), with the use of a novel transfection enhancer that facilitates endosomal escape and nuclear trafficking, the efficiency of gene delivery was increased significantly 24 h post transfection. The present study demonstrated an appropriately optimized protocol that enabled the utility of a novel cationic polymer blend with a mixture of fusogenic lipids and a histone deacetylate inhibitor in non-viral transfection, thereby providing an attractive alternative to costly commercial gene carriers.

Despite the significant disadvantages associated with clinical applications of viral gene carriers, they remain as the mainstream use in most gene-based applications [1–3]. Non-viral gene delivery serves as attractive alternatives due to their advantages that include reduced immunogenicity, the ability to accommodate large size of transgenes, improved safety, and scalability [4,5]. However, the poor transfection efficiency of non-viral carriers severely limits its applications [4]*.* Recently, bio-based polymers are attracting increased attention as some biomolecules carry specific features that benefit biological or cellular applications [6]. Polyplexes formed with combined natural and synthetic polymers were shown to exhibit lower cytotoxicity [7,8]. Natural polymeric backbone carriers have been explored for transfection that include cyclodextrin [9,10], chitosan [11–13], and pullulan [14,15].

Several studies have reported increased reactive oxygen species (ROS) after transfection, leading to poor cell fitness [16–18]. We hypothesized that bio-based polymers with functional groups that specifically reduce ROS post transfection would be useful in gene delivery. Lignin has found applications in a wide variety of fields ranging from cosmetics products to surfactant and raw material for flavoring agents. Some advanced properties, such as biodegradability and antioxidant activity, have motivated interest in developing lignin into biomaterials for biomedical applications [19,20]. Lignin serves as an attractive building block for bio-based polymer as it acts as a free radical scavenger [21,22], is biocompatible, and comprises sufficient reactive functional groups.

Our previous study has shown successful decoration of poly(glycidyl methacrylate)-co-poly(ethylene glycol)methacrylate (PGMA-PEGMA) with lignin through atom transfer radical polymerization method (Supplementary Figure S1A) [19]. Lignin-PGEA-PEGMA graft copolymer (LG100) displays a typical ^1^H NMR spectrum of *M*_n_ of 14.8 kDa with the polydispersity of 1.36 (Supplementary Figure S1B). In this work, LG100 spontaneously forms nanoparticles with negatively charged DNA via ionic interaction. The characteristics of LG100 polyplexes have been discussed extensively in our previous work [19]. Briefly, lignin copolymers are capable to compact pDNA efficiently when the N/P ratio was >1.5, demonstrating better condensation capability than PEI (fully retarding pDNA when N/P ratio > 2.5). As expected, increasing N/P ratio resulted in the formation of smaller LG100 polyplexes (Supplementary Figure S3). Taken these data together, we conclude that the lignin copolymers are able to efficiently compact pDNA into small polyplexes. Moreover, we evaluated the gene transfection efficiency of the lignin copolymers by using luciferase in HEK 293T cells. In the present study, we aim to explore other transfection parameters to improve the efficiency of LG100.

Having determined that LG100 performs as well as PEI, which is widely regarded as the gold standard of polymeric gene transfection agents [19,23], we proceeded to optimize the transfection parameters of LG100. As previously shown, the optimal N/P ratios in HEK293T ranges from 20 to 50. To establish a reliable protocol, we tested and compared transfection of LG100 at various amounts of DNA at N/P ratio of 40. HEK293T cells were transfected with a plasmid encoding GFP reporter gene in 24-well tissue culture vessels to monitor the efficiencies of various parameters. Despite the high transfection efficiency, alteration of cell morphology and reduction in cell number were observed with increasing DNA amount (Figure 1). We hypothesized that the cytotoxicity was due to the exposure of cells to excess polymer. Various studies have reported that excess PEI plays a significant role in efficient transfection but also contributes to cytotoxicity in a dose-dependent manner [24–26]. Removal of excess polymers via size-exclusion chromatography (SEC) greatly improved the toxic profile of PEI polyplexes [26]. On the other hand, mild centrifugation at 200–280 ***g*** for 5 min was shown to facilitate the deposition of polyplexes [27,28]. Instead of SEC, we explored the feasibility of mild centrifugation in depositing LG100 polyplexes onto the cell surface and subsequently removing excess polymer. Immediately after centrifugation, the supernatant is replaced with fresh medium. As shown by the Rhodamine-labeled pDNA polyplexes, comparable amounts of LG100 polyplexes were observed on the cells transfected with 4-h incubation or centrifugation methods (Figure 2A). The cytotoxicity of the supernatant collected post centrifugation of transfection mixture further supports our hypothesis (Figure 2B).

**Figure 1:**
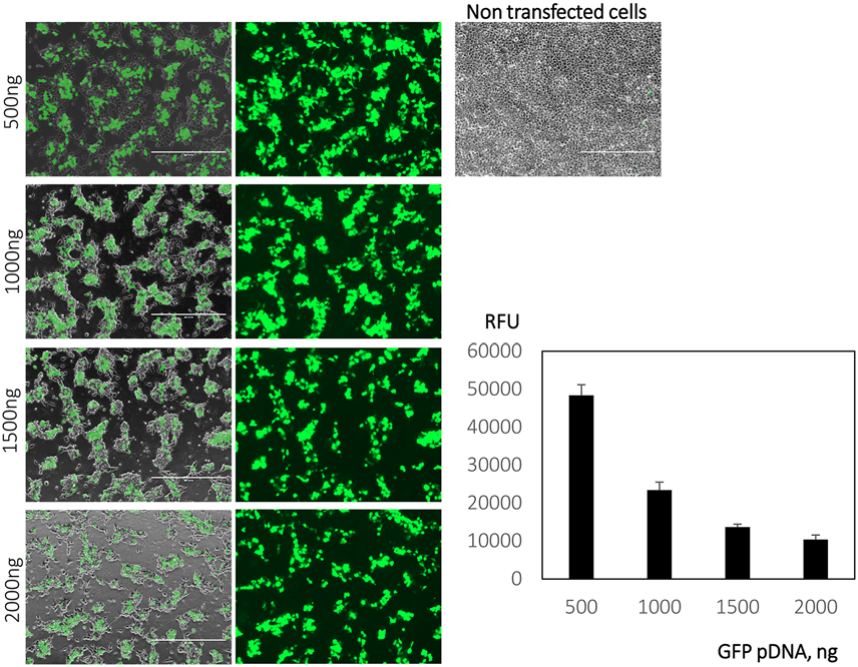
Increasing DNA amount resulted in cytotoxicity HEK293T cells were transfected with pDNA amount ranging from 500 to 2000 ng of GFP expression plasmid. Briefly, the polyplexes were prepared at N/P ratio of 40 by incubating pDNA and LG100 in SF DMEM for 15 min at room temperature. The polyplexes were added to the cell culture and further incubate for 4 h at 37°C. Twenty-four hours post transfection, bright field and fluorescent images were captured at 4× magnification. Scale bar represents 1000 µm. Then, the cell cultures were subjected to microplate reader to measure the GFP expression (RFU) spectrophotometrically. GFP expression was measured (*E*_x_ = 475/*E*_m_ = 509) at nine areas of each biological replicates (*n*=3). Graph represents mean of RFU ± standard error of the mean (SEM).

**Figure 2:**
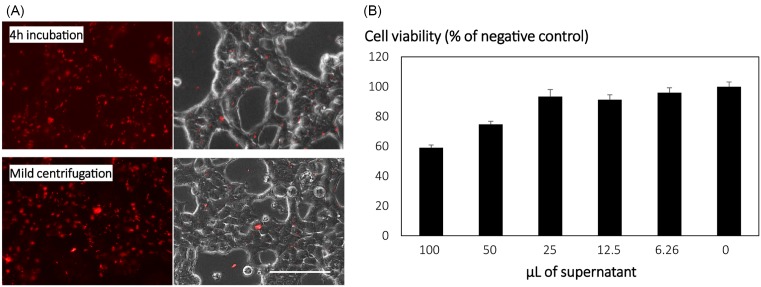
Deposition of LG100 through mild centrifugation HEK293T cells were transfected with 500 ng of Rhodamine-labeled pDNA. LG100 polyplexes were prepared at N/P ratio of 40 in SF DMEM for 15 min at room temperature. After which, the polyplexes were added to the cell culture and subjected to mild centrifugation at 200 ***g*** for 5 min or incubated for 4 h at 37°C. (**A**) After 4-h incubation, fluorescent and bright field images were captured at 10× magnification. Scale bar represents 100 µm. (**B**) The supernatant were collected to examine the cellular toxicity of the free polymers. Various volumes of supernatant were added to HEK293T cell lines in 96-well culture vessels. Twenty-four hours later, the cytotoxic effects were evaluated qualitatively by standard MTS assay. Conditions without treatment of the supernatant served as negative control that was set as 100%. Graph represents mean ± standard deviation (SD), *n*=6.

As expected, cell viability and morphology of HEK 293T cells remained unchanged in centrifugation-mediated transfection (Figure 3A). However, attempts to prevent exposure of cells to excess polymers so as to reduce toxicity substantially reduced transfection efficiency (Figure 3B). Next, we optimized the transfection efficiency of LG100 DNA complexes prepared by varying the amount of DNA (500–1000 ng) and N/P ratio (30–50). As expected, increasing N/P ratio or DNA amount resulted in increased GFP expression. Highest relative fluorescent unit (RFU) (∼8000) was obtained with conditions where 1000 ng of plasmid DNA was used for complexation (Figure 4A). It is worthy to note that comparable cell density to the negative control was observed with cells transfected with N/P 30 and N/P 40 using 500–1000 ng of plasmid (Figure 4B). At N/P 50, the overall RFU was significantly lower at higher DNA amount, suggesting the reduction in cell number. This observation was confirmed with the images captured after nuclear staining. With the mild centrifugation protocol, these results collectively suggested that the LG100 complexes at N/P 30 or N/P 40 with 1000 ng of DNA served as the best condition for efficient transfection in HEK293T without cytotoxicity.

**Figure 3:**
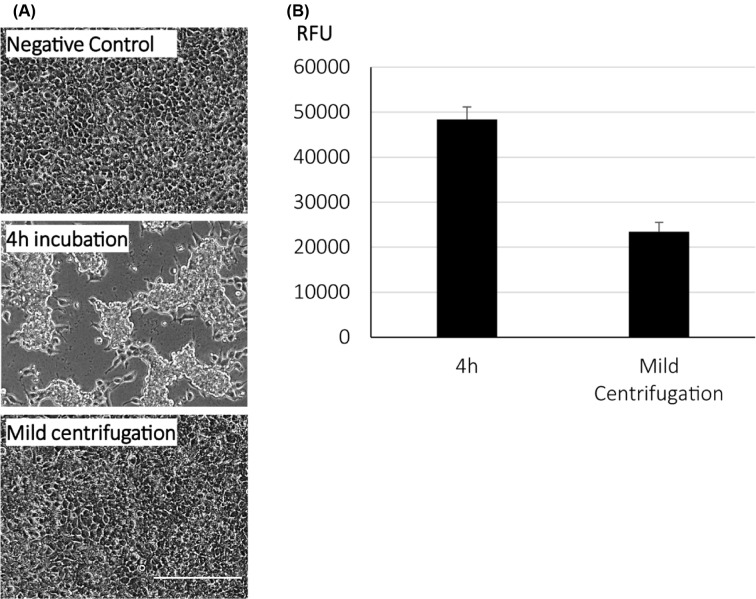
Mild centrifugation reduced transfection induced cellular stress HEK29T cells were transfected with LG100 complexed with 1000 ng of pmaxGFP at N/P = 40. Cells were transfected through 4 h incubation with transfection mixture or mild centrifugation. (**A**) Representative bright field images captured at 48 h post transfection are shown. Scale bar represents 400 µm. (**B**) RFU measurement with Synergy H1 microplate reader. The RFU was recorded with the gain setting at 100. The data shown were the mean ± SEM, *n*=3.

**Figure 4:**
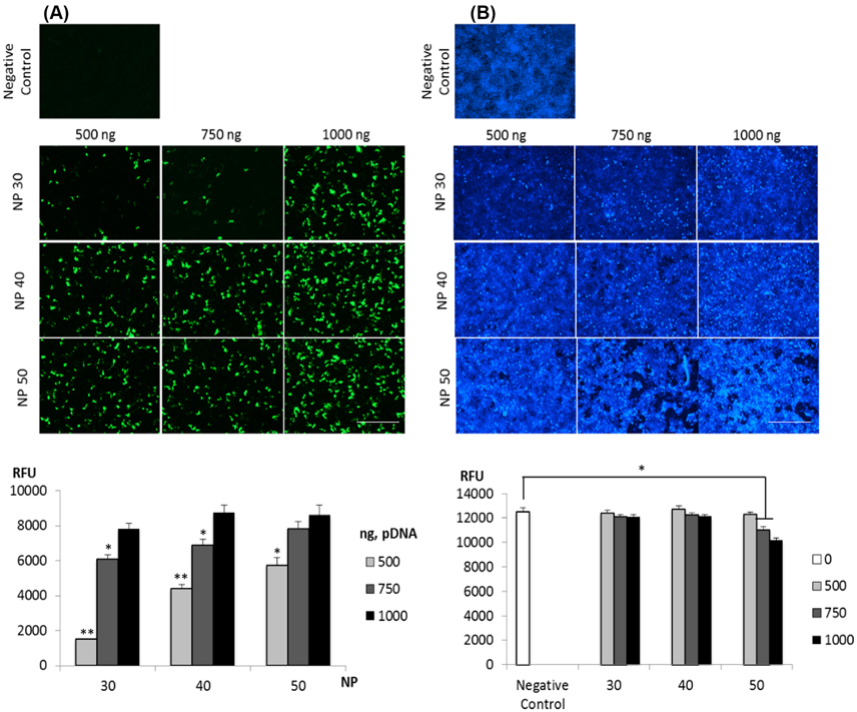
Transfection of HEK293T with LG100 DNA complexes prepared at various DNA amount and N/P ratios LG100 was complexed with 500, 750, or 1000 ng of PMAXGFP at N/P of 30, 40, or 50. The DNA complexes were prepared in SF DMEM. The DNA complexes were added into cells and centrifugated for 5 min. After which, the transfection mixture was replaced with fresh culture media and further incubated for 24 h. Next, cells were fixed with 4% paraformaldehyde and stained with 1 mg/ml Hoechst 33342. (**A**) GFP fluorescent images were captured and samples were subjected to RFU measurement with the microplate reader at *E*_x_/*E*_m_ 475/510. (**B**) DAPI fluorescent images were captured and RFU was measured with microplate reader adjusted for *E*_x_/*E*_m_ 361/467. The non-transfected cells serve as negative control. Representative images are shown. Graph displayed average ± SEM (*n*=2). Scale bar represents 400 µm. Statistical significance of RFU between cells exposed to DNA complexes were obtained using two tailed Student’s *t*-test; *P<0.05; **P<0.0005.

We speculated that the reduction of transfection efficiency in the centrifugation method (Figure 3B) was likely to be due to the lack of free polymers in facilitating the intracellular trafficking of LG100 polyplexes [26,29,30]. In view of the toxicity of LG100 free polymers, we opted for other chemical-based enhancers to ensure that the quality of cells is not compromised post transfection. We have previously explored the effects of various enhancers in improving gene delivery including chloroquine [31], PLUS reagent™, and fusogenic peptides [32]. In all cases, these reagents did not significantly improve the transfection efficiencies in differentiated neuronal cells [33]. Our previous study in unraveling the mechanisms in intracellular trafficking of polyplexes resulted in the development of a transfection enhancing method [33]. The composition of matter is a mixture of reagents composed of fusogenic lipids and a histone deacetylate inhibitor (HDACi). Here, we tested the potential of these reagents in enhancing transfection efficiency following mild centrifugation of the LG100-pDNA polyplexes. Evidently, the addition of fusogenic lipids and HDACi increased total GFP expression (RFU) in HEK293T transfected with 1 µg of pDNA at N/P 30 and 40 by 2.7- and 1.9-fold, respectively (Figure 5A,B). The number of GFP+ cells improved significantly in the presence of these reagents (Figure 5C). It is worthy to note that the use of these reagents did not affect the cell viability. In all conditions with the fusogenic lipids and HDACi and/or polyplexes treatment, the percentage of viable cells was comparable with the control condition (no transfection, in the absence of the reagents) (Figure 5D). To examine the usefulness of transfection system proposed herein, a comparative study of LG100 with commercial reagents, Lipofectamine 2000 (Invitrogen) and Polyfect (Qiagen), was performed in HEK293T and U-20S cell lines (Figure 6). Interestingly, the transfection efficiencies of various gene carriers were highly cell dependent. LG100 was inferior to Lipofectamine 2000 and polyfect in HEK293T but outperformed its counterparts in U-20S cell line. Unlike LG100, it is interestingly to note that increasing DNA amount did not further improve transfection of the commercial reagents in U-20S transfection.

**Figure 5:**
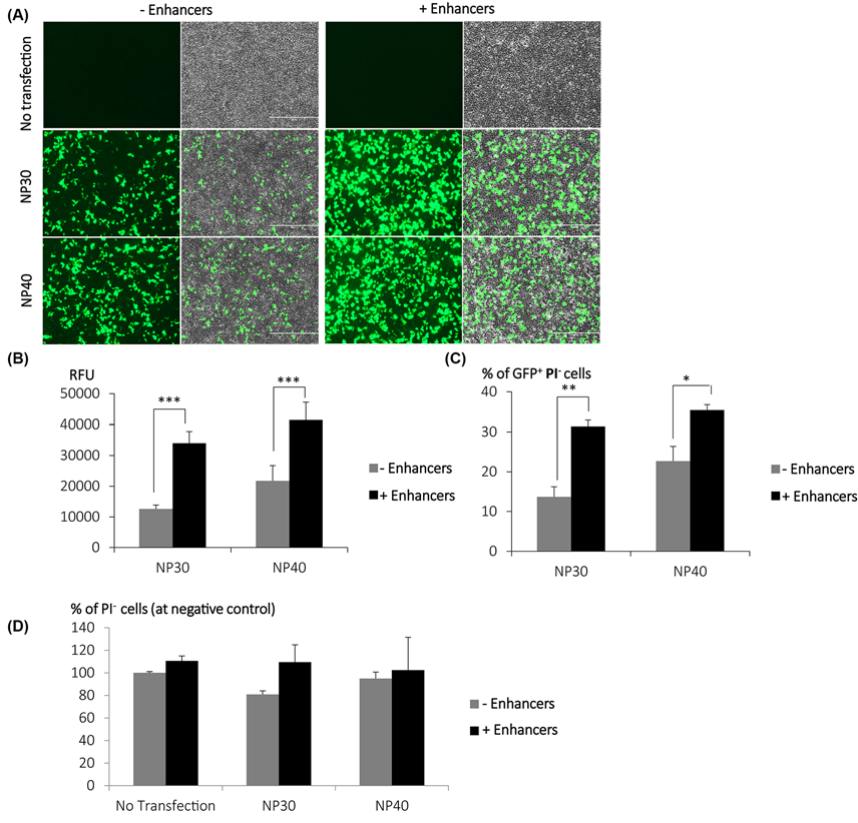
Transfection of HEK293T in the presence or absence of enhancers LG100 was complexed with 1000 ng of PMAXGFP at N/P of 30 and 40. The complexes were prepared as described in Figure 2. After centrifugation, the transfection mixture was replaced with fresh medium with/without enhancers. (**A**) GFP fluorescent images were captured. Representative images are shown. Scale bar indicates 400 µm. (**B**) The samples were then subjected to RFU measurement with the microplate reader. Graph displayed average of RFU (nine readings per well) ± SEM (*n*=3). The cells were trypsinized, pelleted and resuspended in 1× PBS containing 1 µg/ml Hoechst stain 33342 and propidium iodide. The suspension cells were then subjected to (**C**) transfection efficiency and (**D**) cell viability analysis through the Nucleocounter cell counting system. Graph displayed average ± SD (*n*=3). Statistical significance of RFU between conditions with or without enhancers treatment was obtained using two tailed Student’s *t*-test; *P<0.1; **P<0.01; ***P<0.0001.

**Figure 6:**
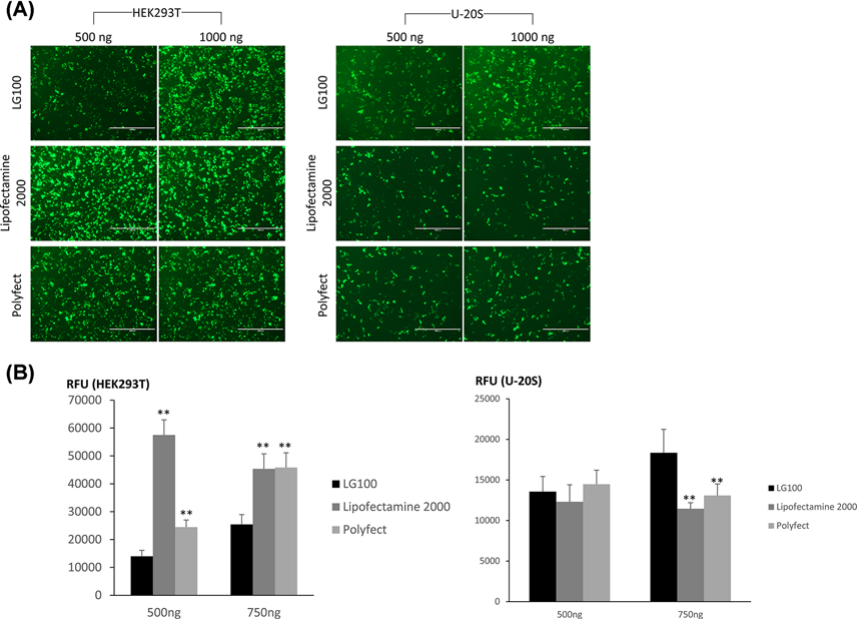
Transfection commercial reagents and LG100 in HEK293T and U-20S LG100, Lipofectamine 2000 (Invitrogen) and Polyfect (Qiagen) were complexed with 500 or 750 ng of PMAXGFP. LG00 polyplexes were prepared at N/P ratio of 40 as described in Figure 2. The complexes of Lipofectamine 2000 and Polyfect were prepared according to manufacturer’s instruction. Twenty-four hours post transfection, (**A**) GFP fluorescent images were captured. Representative images are shown. Scale bar indicates 1000 µm. (**B**) The samples were then subjected to RFU measurement with the microplate reader. Graph displayed average of RFU (nine readings per well) ± SEM (*n*=3). Statistical significance of RFU between LG100 with the commercial agents was obtained using two tailed Student’s *t*-test; **P<0.001.

In summary, the present study demonstrated a facile approach to improve cell viability and yet achieved high transfection efficiency of cationic polymer-based transfection. Understanding the cause for cellular toxicity of a cationic polymer is crucial in developing an efficient transfection method without affecting the quality of cells. Unlike transfection with the conventional protocol, the cell morphology and cell number of HEK293T cell line in the presence of the fusogenic lipids and HDACi remained unaffected. Modification of the transfection protocol has further accentuated the potential application of LG100 as a gene delivery carrier.

## Methods and materials

### Cell culture

HEK293T (ATCC) cells were cultured and maintained in Dulbecco’s Modified Eagle Medium (DMEM) supplemented with 10% FBS and 1% Pen/Strep. U-20S cells (ATCC) were cultured and maintained in McCoy’s 5A medium supplemented with 10% FBS and 1% Pen/Strep.

### Transfection procedure

Twenty-four hours prior to transfection, 200,000 HEK293T cells were plated in each well (24-well plate format, NUNC). Plasmid DNA expressing EGFP (pmaxGFP, Lonza) was purified according to manufacturer’s instruction (Geneaid Biotech, Taiwan). The purified pDNA was then dissolved in Tris-EDTA (TE) buffer and its concentration was obtained by absorption measurement with Nanodrop (ThermoScientific). The pDNA solution was prepared by addition of pDNA into the serum-free Dulbecco’s Modified Eagle Medium (SF DMEM). Next, the polymer solution (in TE buffer) was added dropwise into the pDNA solution, according to the desired molar ratios of nitrogen (N) in polymer to phosphate (P) in pDNA (i.e. N/P ratios). The polymer/DNA mixture was vortexed and incubated at room temperature for 20 min. The transfection mixture was added to cell culture, with or without centrifugation (280 ***g*** for 5 min) at the end of incubation. The transfection mixture was then replaced with culture media and cells were further incubated for 24 h.

To improve transfection, DOPE/CHEMS and Tubastatin A were used. DOPE/CHEMS and Tubastatin A were purchased from Avanti Polar and Bio Vision, respectively. For each reaction in 24-well plate format, 24 µg of DOPE/CHEMS and 10 µM Tubastatin A was used (WO2014070111 A1).

### GFP fluorescent analysis

The fluorescent images and bright field of the transfected HEK293T cells were captured with EVOS FL Cell Imaging System (ThermoScientific) at 10× magnification. The RFU was measured using Synergy H1 Multi-Mode Reader (Biotek). The measurement parameters were predetermined with *E*_x_/*E*_m_ at 485/585 nm and gain at 100, capturing RFU from nine positions in each well.

### Transfection efficiency analysis

The percentage of GFP positive cells was quantified through the Nucleocounter NC-3000 (ChemoMetec) after incubation for indicated periods. At the end of transfection, the cells were trypsinized, centrifuged and re-suspended in PBS containing cell staining reagent (1 µg/ml of Hoechst stain 33342 and 0.5 µg/ml of propidium iodide). Hoechst stain 33342 and propidium iodide were purchased from Life Technologies. At least 1000 cells were analyzed per sample.

### Cell number analysis

To compare the remaining cell number in the cell culture after transfection with polyplexes at various N/P ratios and DNA amount, HEK293T cells were fixed by incubation in 4% paraformaldehyde for 20 min. After which, the cells were stained with 1 µg/ml of Hoechst stain 33342 in 1× PBS for 5 min. Cells were washed once with 1× PBS and subjected to RFU measurement with the Synergy H1 Multi-Mode Reader. The RFU was obtained with predetermined setting of *E*_x_/*E*_m_ at 485/585 nm and gain at 100, capturing RFU from nine positions in each well. The percentage of viable cells was quantified through the Nucleocounter. The sample preparation has been described in section on transfection efficiency analysis. The software provides the information on the number of PI^−^ cells/0.8 µl of samples. This enables the calculations of the % of viable cells.

### Cell viability assay

Six biological replicates of HEK293T (40,000 cells per well) were plated into 96-well plates. Twenty-four hours later, culture medium was replaced for medium containing various volume of supernatant collected post centrifugation of the transfection mixture. One to five days later, plates were subjected to the CellTiter 96 Aqueous One Solution Cell Proliferation Assay (Promega). The colorimetric read out was measured spectrophotometrically at 490 nm. Results were expressed as the percentage of cell viability, in relative to cells in condition without treatment.

## Supporting information

**Figure S1:** (A) Schematic diagram illustrating the synthesis steps of the Lignin-PGEAPEGMA copolymer (LG100), (B) ^1^H of NMR spectrum of LG100

**Figure S2:** (A) TGA and (B) DSC curves of the Lignin-PGEA-PEGMA copolymer (LG100)

**Figure S3:** (A) Electrophoretic mobility of pDNA in the complexes of the LG100 at various N/P ratios. (B) Particle size of polymer/pDNA complexes at various N/P ratios.

## Abbreviations

DMEM: Dulbecco’s Modified Eagle Medium
HDACi: histone deacetylate inhibitor
pDNA: plasmid DNA
RFU: relative fluorescent unit
ROS: reactive oxygen species
SEC: size-exclusion chromatography
