# Supplementary material for: Enhanced transfection of a macromolecular lignin-based DNA complex with low cellular toxicity
Source: Biosci Rep. 2018 Nov 30;38(6):BSR20181021. doi: 10.1042/BSR20181021 (PMC6265617; doi:10.1042/BSR20181021)
Supplement: Supplementary file 1 [file bsr20181021_Supp1.pdf]

Supporting Information

Enhanced transfection of a macromolecular lignin–based DNA complex with low cellular toxicity

Yoon Khei Ho[1*], Dan Kai[2*], Geraldine Xue En Tu[1], G. Roshan Deen[4], Heng Phon Too[1*], Xian Jun Loh[2,3*],

[1] Department of Biochemistry, National University of Singapore, 8 Medical Dr, Singapore 117596

[2]Institute of Materials Research and Engineering, A*STAR (Agency for Science, Technology and Research), 3 Research Link, Singapore 117602

[3]Department of Materials Science and Engineering, National University of Singapore, 9 Engineering Drive 1, Singapore 117576

[4]Soft Materials Laboratory, Natural Sciences and Science Education, National Institute of Education, Nanyang Technological University, Singapore

E-mail: bchhyk@nus.edu.sg (Y.K. HO), kaid@imre.a-star.edu.sg (D. Kai); lohxj@imre.a-star.edu.sg (X.J. LOH), bchtoohp@nus.edu.sg (H.P. TOO)

Synthesis of Lignin-PGEA-PEGMA graft copolymer via ATRP The Lignin-PGMA-PEGMA polymer were synthesized via ATRP. First, alkali lignin (Sigma) was modified by 2-bromoisobutyryl bromide through esterification reaction in the presentation of triethylamine, and the modified lignin (lignin-Br) was collected as macroinitiator for ATRP. Next, Lignin-Br (130.0 mg, 0.3 mmol of initiation site), glycidyl methacrylate (4.26 g, 30.0 mmol), PEGMA (0.99 g, 0.9 mmol), 1,1,4,7,10,10-Hexamethyltriethylenetetramine (69.0 mg, 0.3 mmol) were introduced into a dry flask containing 20 mL of degassed acetone. After degassing the mixture by bubbling nitrogen for 20 min, CuBr (43.0 mg, 0.3 mmol) was added into the mixture under a nitrogen atmosphere. The polymerization was allowed to proceed under continuous stirring at room temperature for 4 h. The final tan mixture was diluted with t*etrahydrofuran* and passed through a short neutral Al2O3 column with t*etrahydrofuran* as eluent to remove copper catalyst. The resulting eluate solution was concentrated to 10 ml and percipitated with 1000 ml hexane. The final product Lignin-PGMA-PEGMA was dried under vacuum at 40 °C for overnight. Next, 0.2 g of Lignin-PGMA-PEGMA was dissolved in 8 mL of dimethylformamide, following by adding 5 mL of ethanolamine and 1 mL of TEA. The reaction mixture was stirred at 40 °C for 5 days. Then, the reaction mixture was precipitated with 500 mL of diethyl ether twice. The crude products were re-dissolved in 10 mL of deionized water and dialyzed against deionized water with dialysis membrane (MWCO, 1000 Da) at room temperature for 2 days. The final products (LG100) were obtained by freeze-drying. Characteristic peaks of PEGMA were shown at 3.3 (a) and 3.7 ppm (b) corresponding to methyl and methylene protons from PEGMA (Figure S1). The peak c at 4 ppm corresponds to CH_2-O-C=O protons, and the peak h at 2.7 ppm was attributed to the methylene protons from NH-CH2. The lignin peak from methoxy protons is at 3.7 ppm which was overlapped with the PEGMA peak. By calculation (according to M_n of lignin = 5 kDa and NMR results), such polymer contained 33.8 wt% of lignin and 46.9 wt% of PGEA.

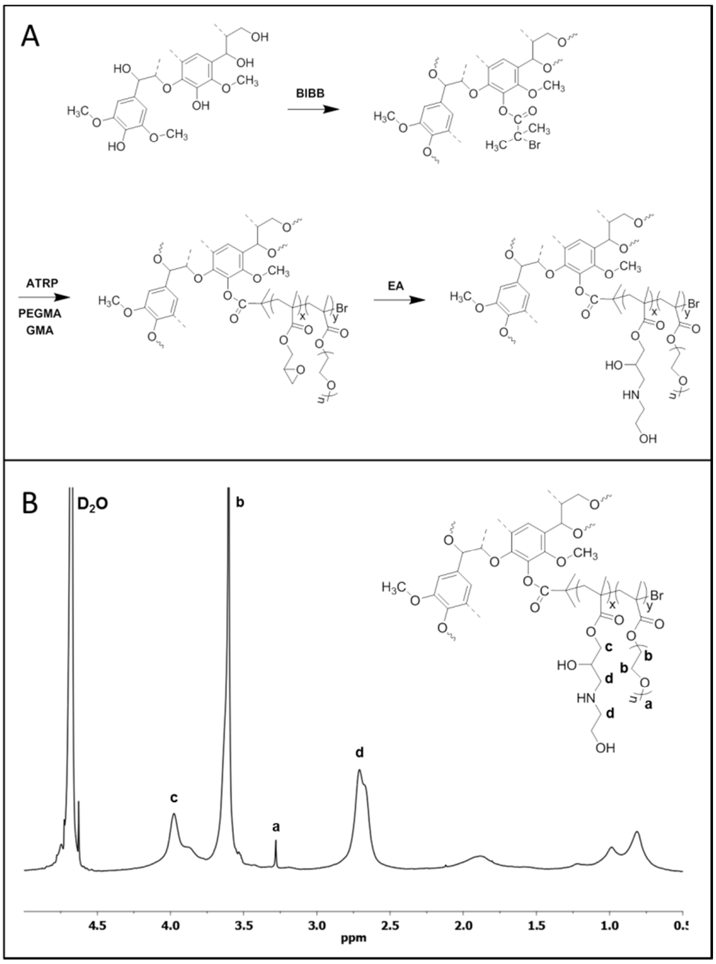

Figure S1. (A) Schematic diagram illustrating the synthesis steps of the Lignin-PGEA-PEGMA copolymer (LG100), (B) ^{1}H of NMR spectrum of LG100

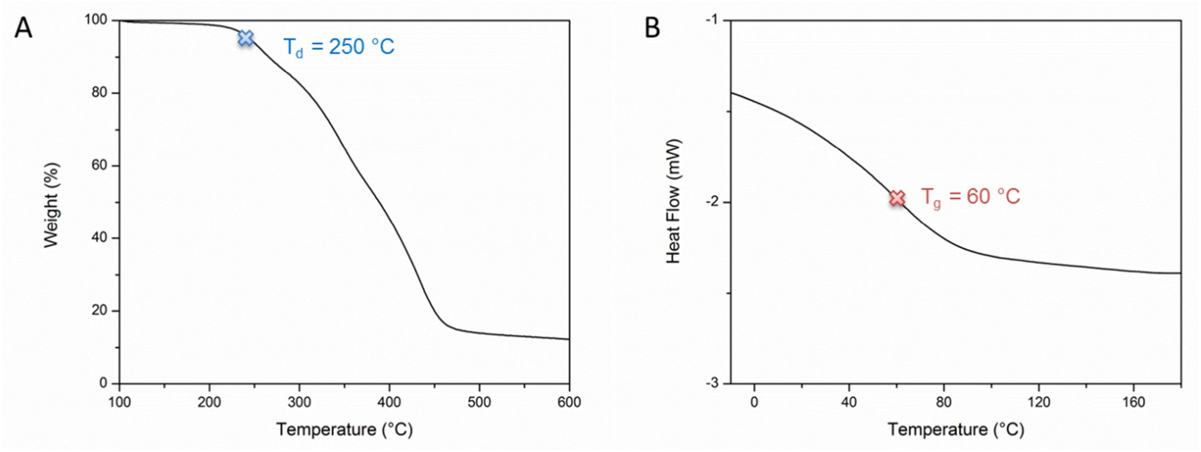

Figure S2. (A) TGA and (B) DSC curves of the Lignin-PGEA-PEGMA copolymer (LG100)

The thermal properties of LG100 were evaluated by thermogravimetric analysis (TGA) and differential scanning calorimeter (DSC). As shown in Figure S2A, LG100 showed 5% of the weight loss (thermal decomposition temperature, T_d) at 250 °C, which is similar to the T_d of lignin (260 °C). Such copolymer remained 12.3% of its original weight at 600 °C. As PGEA and PEGMA segments were completely decomposed at 500 °C, the residue of LG100 was the charred lignin. As shown in Figure S2B, DSC analysis exhibited that amorphous LG100 displayed a glass transition temperature (T_g) at 60 °C, which is remarkably lower than the T_g of raw lignin (164 °C). In the previous studies, it was demonstrated that the thermal properties of lignin-based copolymers were highly relative to the grafted polymer side chains.[1] In this case, the grafted PGEA-PEGMA side chains significantly reduced the T_g of the copolymer, and its T_g could be tuned by varying the chain lengths or the ratios of PGEA:PEGMA.

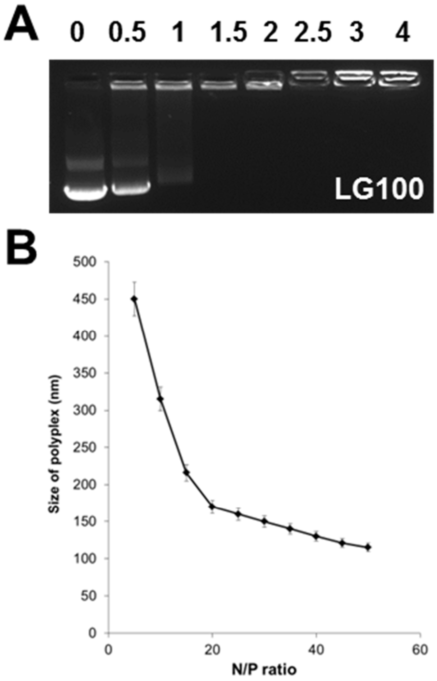

Figure S3. (A) Electrophoretic mobility of pDNA in the complexes of the LG100 at various N/P ratios. (B) Particle size of polymer/pDNA complexes at various N/P ratios.

[1] S. Sen, S. Patil, D. S. Argyropoulos, *Green Chemistry* **2015**.
